# Fatty Acid Diets: Regulation of Gut Microbiota Composition and Obesity and Its Related Metabolic Dysbiosis

**DOI:** 10.3390/ijms21114093

**Published:** 2020-06-08

**Authors:** David Johane Machate, Priscila Silva Figueiredo, Gabriela Marcelino, Rita de Cássia Avellaneda Guimarães, Priscila Aiko Hiane, Danielle Bogo, Verônica Assalin Zorgetto Pinheiro, Lincoln Carlos Silva de Oliveira, Arnildo Pott

**Affiliations:** 1 Graduate Program in Biotechnology and Biodiversity in the Central-West Region of Brazil, Federal University of Mato Grosso do Sul, Campo Grande 79079-900, Brazil; machatedavidjohanemachate@yahoo.com.br (D.J.M.); arnildo.pott@gmail.com (A.P.); 2 Graduate Program in Health and Development in the Central-West Region of Brazil, Federal University of Mato Grosso do Sul, Campo Grande 79079-900, Brazil; pri.figueiredo92@gmail.com (P.S.F.); gabi19ac@gmail.com (G.M.); priscila.hiane@ufms.br (P.A.H.); daniellebogo@hotmail.com (D.B.); veronica.azp@outlook.com (V.A.Z.P.); 3 Chemistry Institute, Federal University of Mato Grosso do Sul, Campo Grande 79079-900, Brazil; lincoln.oliveira@ufms.br

**Keywords:** health, short-chain fatty acids, intestinal bacteria, hypertension, inflammatory diseases, diabetes

## Abstract

Long-term high-fat dietary intake plays a crucial role in the composition of gut microbiota in animal models and human subjects, which affect directly short-chain fatty acid (SCFA) production and host health. This review aims to highlight the interplay of fatty acid (FA) intake and gut microbiota composition and its interaction with hosts in health promotion and obesity prevention and its related metabolic dysbiosis. The abundance of the Bacteroidetes/Firmicutes ratio, as Actinobacteria and Proteobacteria species are associated with increased SCFA production, reported high-fat diet rich in medium-chain fatty acids (MCFAs), monounsaturated fatty acids (MUFAs), and n–3 polyunsaturated fatty acids (PUFAs) as well as low-fat diets rich in long-chain fatty acids (LCFAs). SCFAs play a key role in health promotion and prevention and, reduction and reversion of metabolic syndromes in the host. Furthermore, in this review, we discussed the type of fatty acids and their amount, including the administration time and their interplay with gut microbiota and its results about health or several metabolic dysbioses undergone by hosts.

## 1. Introduction

Fatty acids (FAs) are the principal components of triacylglycerols found in oils and fats, which are the second primary source of dietary energy for humans [1]. Several FAs are obtained from different types of foodstuff and can be affected during their processing, storage, and cooking and various eating habits. The majority of FAs dietary intake (>95%) is available to the bloodstream through efficient processes of digestion and absorption [2].

FAs furnish energy (9 kcal per gram corresponding to 20%–35% of total calorie intake in adults) [3]; carry fat-soluble vitamins (A, D, E, and K) [4]; constitute the cell-membrane phospholipids; and act on its fluidity and signaling [2], immune system regulation, blood clots, and cholesterol metabolism [5].

On the other hand, fat production and accumulation in the body can be related to calories furnished by unbalanced FA intake and expended quantities, related to a lack of physical activities, genetic predisposition, and pathways involving metabolites and hormones [6,7,8].

**Figure 1:**
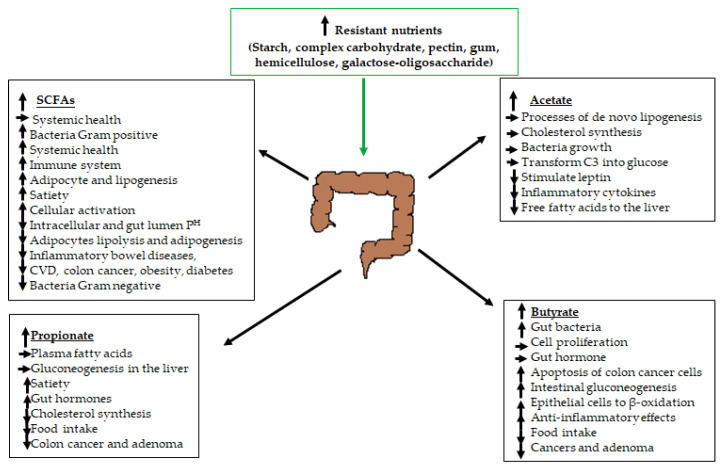
The role of the gut microbiota in short-chain fatty acid (SCFA) production and their benefits to human physiology regulation, which have contributed greatly in health promotion and disease prevention. Abbreviation: ↑ = significant increase; ↓ = significant decrease; → = stable performance C3 = propionate; and CVD = cardiovascular diseases.

Furthermore, these dysfunctions can be associated with unbalanced microbiota composition in the host gut, which is the complex tract through which food passes during a lifetime and has found an abundant and dynamic population of microbiota [9]. Thus, the gut is home to about 100 trillion organisms, with 35,000 species of bacteria, of which small the intestine presents 10^7^–10^8^ and the large intestine presents 10^10^–10^11^ cells per mL of contents [10,11]. This microbiota compound is mainly anaerobic, in which 98% is constituted by phyla Bacteroidetes (9%–42%) (*Porpyromonas* and *Prevotella*), Firmicutes (30–52%) (*Ruminococcus, Clostridium,* and *Eubacteria*) and Actinobacteria (1–13%) (*Bifidobacterium*) and in which 2% is constituted by phylum Lactobacillae (2%) (*Streptococci* (2%) and *Enterobacteria* (1%)) [12,13]. The most crucial gut microbiota activity is involved with short-chain fatty acids (SCFAs), produced by fermentation, which is represented by Ruminococcaceae and *Eubacterium* in the order Clostridia, classes Clostridia and Firmicutes, from prebiotics: polysaccharides (resistant starches, hemicellulose, pectins, and gums), oligosaccharides, proteins, peptides, and glycoproteins [14,15,16]. The SCFAs are a group that presents 1–6 saturated carbons in their structures. The most relevant SCFAs produced are acetate > propionate ≥ butyrate [17]. Propionate is abundantly synthesized by Bacteroidetes and Negativicutes, utilizing succinate [18]. Butyrate is broadly produced by Clostridial clusters IV, XIVa, and XVI (Firmicutes) through the butyrate kinase or butyryl-coenzyme A (CoA): acetate CoA-transferase pathways, and this last pathway provides a high quantity of acetate [19]. The beneficial effects of the SCFAs produced by the gut microbiota are summarized in Figure 1.

The SCFAs produced in the colon are immediately absorbed and furnish energy for colonocytes, and the remaining SCFAs are immediately incorporated into the hepatic portal vein by passive diffusion and active transport mechanisms and contribute to the optimal function of several organs [20,21,22]. Therefore, studies have demonstrated that the energy furnished to the host from diet intake is associated with modulation of the gut microbiota composition and leads to SCFA production [23,24,25,26]. Also, FA diet has contributed to health promotion and disease prevention, including obesity and its related disorders [27,28,29,30].

Obesity represents a consequence of abnormal fat accumulation in the body, resulting in high energy, which may lead to a pro-inflammatory response, and culminating at several disorders [31,32,33], such as insulin resistance and inflammatory diseases.

The objective of this review is to provide an overview of fatty acid intake and gut microbiota composition for host health promotion and obesity prevention and its related metabolic dysbioses (e.g., coronary heart diseases and type 2 diabetes mellitus) through the compilation of several scientific articles published in the last five years related to studies with animal models and human subjects.

## 2. Medium-Chain Fatty Acids

Medium-chain fatty acids (MCFAs) are a group that presents 7–12 saturated carbons in their structures. The most common MCFAs are caprylic (C8:0), capric (C10:0), and lauric (C12:0) acids [34]. The common diet sources of caprylic, capric, and lauric acids are coconut, palm kernel, and human milk with 5–8%, 6–7%, and 48–58%, respectively [35,36,37,38]. MCFA digestion and absorption occur in the stomach, catalyzed by lingual and gastric lipases, solubilized in the aqueous phase of the intestinal contents, absorbed bounded to albumin, and transported to the liver via the portal vein [39,40,41]. These acids do not need carnitine shuttle to enter mitochondria; however, they increase the energy spent, regulate protein activation, reduce adiposity and preserve insulin action in muscle and fat, induce satiety, increase mucosal microvillus enzymes activity in the small intestine, elongate to long-chain fatty acids, and resynthesize triglycerides [42,43,44,45]. MCFAs have shorter biological half-time and higher stability to lipoperoxidation [34]. Considering the lack of scientific evidence that address human studies with MCFAs, the effects of MCFAs-rich diet consumption on gut microbiota on obesity and its related diseases that occur in animal model studies are summarized in Table 1.

Several studies on MCFAs reported that the increase of Bacteroidetes and the decrease of Firmicutes and Proteobacteria in mice gut consequently lowered the inflammation and obesity effects [46]. Furthermore, the increase of the Bacteroidetes to Firmicutes ratio as well as the abundance of *Ruminococcaceae*, *Bifidobacterium*, and *Lactobacillus* are associated with SCFA production [15,50,51]. Moreover, these bacteria are correlated with reducing effects of obesity, inflammatory bowel disease (IBD), type 2 diabetes mellitus (T2DM), and cardiovascular diseases (CVD) in the hosts [51,52,53,54,55]. *Bifidobacterium* and *Lactobacillus* are predominantly abundant in the human gut during early life, producing lactate and acetate acids protecting the hosts against enter-pathogenic agents [50,51,56,57]. The natural sources of MCFAs are human milk (9–15%) and virgin coconut oil (61%), presenting higher composition compared with infant formula (8–42%) [37,38,58,59].

However, diets rich in coconut oil ≥25% administrated to healthy female animal models for 8 or 10 weeks showed obesity and its related dysfunction effects and increase of *Allobaculum, Clostridium, Lactobacillus*, *Staphylococcus*, and the Firmicutes to Bacteroidetes ratio in their guts [48,49,52,60].

## 3. Long-Chain Fatty Acids

Long-chain fatty acids (LCFAs) are a group that presents 13–18 saturated carbons in their structures. The LCFAs in the diet are myristic (C14:0), palmitic (C16:0), and stearic (C18:0) acids [41]. The primary dietary sources of myristic acid include human milk, palm olein, and coconut (8–20%) [37,38,61]. Palmitic acid (PA) is commonly found in olive, human milk, cottonseed, and palm olein (20–47%) [37,38,61,62]. Stearic acid occurs in pumpkin, sesame, and human milk (6–7%) [37,38].

Therefore, LCFAs represent 80–90% of total saturated fatty acid food intake [34], between 20–30 g per day corresponding to PA [63]. However, PA intake (exogenous) is counterbalanced by PA endogenous biosynthesis via de novo *lipogenesis* (DNL), crucial to maintaining cell membrane fluidity and insulin sensitivity [64]. In normal physiology conditions, PA accumulation is prevented by enhanced Δ-9 desaturation to palmitoleic acid (16:1 n-7) and/or elongation to stearic acid and/or Δ-9 desaturation to oleic (18:1 n-9) and then elongation to eicosenoic acid (20:1 n-9) [34,65]. The effects of LCFA-rich diet consumption on gut microbiota composition in animal models are summarized in Table 2.

In general, the increase of the Bacteroidetes to Firmicutes ratio was due to low fatty acid diets (7% of energy), and high fatty acids (25% of energy) of LCFAs was reported for healthy men and women (21–65 years old) [74]. Additionally, the main genera recorded by several studies are represented by *Blautia, Clostridium, Coprococcus, Dialister, Lachnospira, Lactococcus, Lachnobacterium, Phascolarctobacterium, Roseburia, Ruminococcus* (Firmicutes), *Bacteroides*, *Paraprevotella, Parabacteroides,* and *Prevotella* (Bacteroidetes), correlated with SCFA production, obesity, and its related metabolic dysbiosis reduction [75,76,77,78,79,80,81].

Additionally, another gut microbiota feature is related to the most abundant Firmicutes in the intestine of healthy subjects and followed by relatively increasing Bacteroidetes [82,83]. This behavior is maintained by equilibrated amounts of energy intake and expenditure by the host, which play a key role to keep the symbiotic relationship between gut microbiota and host [84]. Thus, this harmonic relationship between the host and gut microbiota can allow the increase of SCFA production (acetic, propionic and butyric acids) which are crucial to the homeostasis and diseases of the host [9,85].

However, the increase of the Firmicutes to Bacteroidetes ratio, including Actinobacteria, was recorded with LCFA-rich diets (34–72% of energy) fed to healthy animal models. Additionally, increased effects of obesity, adipose tissue, plasma cholesterol, total cholesterol, weight gain, hypertension, insulin resistance, inflammatory bowel diseases (IBD), nonalcoholic steatohepatitis (NASH) in the studied subjects occurred [66,67,68,69,72]. Obesity and its related metabolic syndromes are associated with increase of *Desulfovibrio* and *Bilophila wadsworthia* (Proteobacteria) and decrease of *Bifidobacterium* spp. (Actinobacteria) [70,71].

Therefore, higher caloric intake and lower energy expenditure by animal models and human subjects show increasing Firmicutes abilities for energy extraction from diet and SCFA (acetate and butyrate) production and consequently elevating mass weight gain of the host and obesity by fat accumulation in adipocyte tissue [86,87]. Additionally, decreasing Bacteroidetes at 50% compared with the Firmicutes ratio, including the abundance of Actinobacteria and Proteobacteria, is correlated with obesity and its related metabolic dysbioses [83,88,89,90].

## 4. Monounsaturated Fatty Acids

Monounsaturated fatty acids (MUFAs) are an unsaturated group with one double bond in their structures. The MUFAs include palmitoleic (C16:1 n-7), oleic (C18:1 n-9), and eicosenoic (C20:1 n-9) acids [37]. The MUFAs are endogenously obtained by Δ-9 desaturation, palmitoleic from palmitic acid and oleic from stearic acid, and by elongation of oleic to eicosenoic acid [34]. The MUFAs are obtained through ingestion; oleic acid is the most representative with 25–71% in safflower, sesame, pumpkin seed, rice bran, human milk, rapeseed, olive, and peanut [37,38] and with eicosenoic acid with 7–17% in wheat germ, rapeseed, and hemp [37].

MUFA consumption is associated with reduced effects of obesity and its related metabolic syndromes [91,92,93]. Furthermore, these health beneficial effects demonstrated by MUFAs result from their apolipoproteins (E and C-III) that present a high affinity for the hepatic receptors and rapidly activate synthetic and catabolic pathways for triacylglycerol-rich lipoprotein metabolism [94,95]. Moreover, the consumption of MUFAs-rich diet showed positive health effects, e.g., extra virgin olive oil increased the gut microbiota diversity of healthy and unhealthy animal models, including humans under risk of metabolic syndrome [74,96,97]. Effects of MUFA-rich diet consumption on gut microbiota composition in animal models are summarized in Table 3.

The increased Bacteroidetes to Firmicutes ratio, including *Bifidobacterium* spp. (Actinobacteria), was recorded for MUFA-rich diet (10–76% of energy) administrated to humans for several weeks (Table 4). The increase in Bacteroidetes and *Bifidobacterium* spp. is correlated with high SCFA (acetic, propionic and butyric acids) production [15,101,102]. Among SCFAs, butyrate is the most important because it is an energy source for colonocytes and, on the other hand, triggers Firmicutes to reduce dietary energy harvest and consequently decreases adipose tissue fat accumulation in hosts [84,86,87,103].

Consequently, MUFA-rich diet shows decreasing effects of obesity, weight gain, insulin resistance, hypertension, body mass index (BMI), and nonalcoholic steatohepatitis (NASH) [73,96,97,100]. SCFAs are crucial biomacromolecular substances utilized for the homeostasis and disease of the host, protecting or reducing the effects of obesity, diabetes, inflammatory bowel diseases (IBD) and cardiovascular diseases (CVD) [9,103,104].

## 5. Polyunsaturated Fatty Acids

The polyunsaturated fatty acids (PUFAs) are an unsaturated group that presents two or up to six double bonds in their structures. PUFAs are essential FAs (cannot be synthesized by human or higher animals’ bodies and are required from dietary intake) constituted by α-linolenic acid (ALA) from the n–3 PUFA family and by linoleic acid (LA) from the n–6 PUFA family [106]. ALA is abundant in flaxseed (53 g), canola (18 g), and soybean oils (7 g). LA is found in soybean (56 g), corn (53 g), canola (19 g), flaxseed (14 g), and safflower oils (12.72 g) [107].

In the body, ALA is converted to eicosapentaenoic (EPA) and docosahexaenoic acids (DHA) through a series of desaturation and elongation reactions and presents effects of anti-inflammation, vasodilation, bronchodilation, and anti-platelet aggregation, and LA follows the same pathways, shares the same enzymes, competes with ALA for its desaturation and elongation processes, is converted to arachidonic acid (ARA), and presents an antagonistic effect to ALA and pathophysiology [106,108,109].

Furthermore, an n–3 PUFA-rich diet is correlated with decreasing or preventing adipose tissue fat accumulation, insulin resistance, inflammation, hypertension, atherosclerosis, obesity, cardiovascular diseases (CVD), and type 2 diabetes mellitus (T2DM) [110,111,112,113]. In contrast, an n–6 PUFA-rich dietary intake is associated with metabolic dysbioses such as obesity, inflammatory bowel diseases (IBD), nonalcoholic steatohepatitis (NASH), and CVD [71,114,115]. Due to competition and antagonistic effects of n–6 against n–3 PUFAs, the recommended balanced dietary ratio of n–6/n–3 intake is 1/1 or 2/1–10/1 [106,116].

Thus, dietary PUFAs play a crucial role in a host specific to gut microbiota composition and in the ability of the production of MUFA-derived metabolites [104,117]. Also, n–3 PUFA intake is related to the abundance of gut microbiota composition and to increasing SCFA production [101,102,118]. Effects of PUFA-rich diet consumption on gut microbiota composition in animal models are summarized in Table 5.

The increased Bacteroidetes to Firmicutes ratio, including Actinobacteria and Proteobacteria, was reported with administration of n–3 PUFAs in a low fat-diet or high-fat diet and of n–6/n–3 PUFA proportions (1/2 or 3/1–11/1) to humans (Table 6). The results demonstrated the decreased effects of obesity, inflammation, weight gain, nonalcoholic steatohepatitis (NASH), and type 2 diabetes mellitus (T2DM) [80,96,118,124]. Furthermore, the abundance of the Bacteroidetes to Firmicutes ratio is correlated with increasing SCFA (acetate, propionate, and butyrate acids) production [85,102]. Butyrate is a substrate for colonocytes, and all SCFAs produced are important to biomacromolecular substances linked to homeostasis and disease of the host [9,106].

However, interestingly, lowering obesity effects were recorded for healthy female genetically modified mice compared with wild-type mice; administrating safflower oil (n–6 PUFA-rich diet) for 21 weeks increased Bacteroidetes to Firmicutes ratio, including Proteobacteria [119]. Inversely, weight gain remained stable with decreased *Helicobacter* and *Clostridiales* in healthy mice (male and female) given n–3 PUFA of fish oil (40% EPA- and 27% DHA-rich diet) for two weeks [120]. *Helicobacter* and *Clostridiales* are related to increasing effects of insulin resistance, low-density lipoprotein-cholesterol (LDL-C), IBD, NASH, T2DM, and CVD [125,126,127].

With regard to diets, n–6/n–3 PUFA proportion at 1/2 demonstrated anti-inflammatory effects on pups with increased *Blautia* (Firmicutes) and decreased Bacteroidetes [121]. *Blautia* is associated with butyrate production and anti-inflammatory effect [75,128]. An n–6/n–3 PUFA proportion of 3/1 to 11/1 in the diet recorded decreased effects of obesity and its related metabolic dysbioses and increased *Allobaculum*, *Isobaculum,* Proteobacteria, and Lachnospiraceae [80,96,101]. Lachnospiraceae and *Allobaculum* are associated with SCFA production [80,129,130]. Besides, *Allobaculum* is related to high-lipoprotein density-cholesterol (HLD-C) production and reduction in obesity effect [131]. Unfortunately, the beneficial effect of *Isobaculum* on health is yet unknown [98].

Other studies on n–6 PUFA-rich diets reported increasing effects of obesity, weight gain, inflammation, and adipose tissue fat accumulation [71,115]. The increase of *Bacteroides, Bifidobacterium,* Lachnospiraceae Proteobacteria, and Clostridiales is related to metabolic dysfunction risks [132,133,134]. Effects of PUFAs on gut microbiota are summarized in Table 6.

## 6. Conclusions

Different types of FA dietary intakes play a crucial role in modifying the composition of gut microbiota, which interplay the health improvement or disease of the host. The consumption of HFD with a predominance of MCFAs, MUFAs, and n–3 (EPA and DHA), including low fat-diet of LCFA dietary intake, increases the beneficial microbiota, mainly the Bacteroidetes to Firmicutes ratio as well as Actinobacteria and Proteobacteria species. These bacterial species are correlated with increasing SCFA production, which prevents and reduces obesity and its related metabolic dysbiosis effects. However, high-fat diets of LCFAs and n–6 PUFA dietary intake present antagonistic effects and show pathologic results to animal models and human studies compared with other types of fatty acids.

## Figures and Tables

**Table 1:** Effects of medium-chain fatty acid intake on gut microbiota composition and metabolic outcomes in animal models.

Host	Diets	Main Outcomes	
		Gut Microbiota	Related Diseases
Mice C57BL/6J (7 weeks old): healthy male [46]	HFD containing 20% (*w*/*w*) rapeseed oil with MCFAs (30%) for 6 weeks	Bacteroidetes↑ *Allobaculum* and Lachnospiraceae (Firmicutes)↓ *Helicobacter* spp. (Proteobacteria)↓	IBD↓ Obesity↓
Wistar rats (10 weeks old): male with induced diabetic [47]	Virgin coconut oil (caprylic, 6.57%; capric, 5.78%; and lauric, 48.51%) for 16 weeks	*Bifidobacterium* (Actinobacteria)↑ *Allobaculum* and *Lactobacullum* (Firmicutes)↑	T2DM↔
Wistar rats: female [48]	HFD (50 or 95%) of Virgin coconut oil (caprylic, 5.22%; capric, 5.41%; and lauric, 51.64%) for 10 weeks	*Bacteroides* and *Prevotella* (Bacteroidetes)↑ *Bifidobacterium* (Actinobacteria)↑ *Lactobacullum* and *Enterocuccus* (Firmicutes)↑ *Clostridium histolyticum* (Firmicutes)↓	IBD↑ adipose tissue↑ NASH↑
Mice C57BL/6N (3 weeks old): healthy female [49]	HFD containing coconut oil 25% and soy oil 0.25% for 8 weeks	*Allobaculum*, *Staphylococcus, Clostridium*, F16, YS2, *Lactobacillus* (Firmicutes)↑ *Deltaproteobacteria* (Proteobacteria)↑ Bacteroidetes↓	Obesity↑ adipose tissue↑ plasma cholesterol↑.

Abbreviation: ↑ = significant increase; ↓ = significant decrease; ↔ = unchanged; IBD = inflammatory bowel disease; HFD = high-fat diet; MCFAs = medium-chain fatty acids; NASH = nonalcoholic steatohepatitis; and T2DM = type 2 diabetes mellitus.

**Table 2:** Effects of long-chain fatty acids intake on gut microbiota composition and metabolic outcomes in animal models.

Host	Diets	Main Outcomes	
		Gut Microbiota	Metabolic
Mice C57BL/6J (3 weeks old): healthy male [66]	LFD palm oil (rich in palmitic acid) (10% kcal) for 3 weeks	Bacteroidetes↑ *Bacilli* and *Clostridium* cluster XI, XVII, and XVIII (Firmicutes)↓.	Adipose tissue↓ weight gain↓ NASH↓ insulin resistance↓
	HFD palm oil (rich in palmitic acid) (45% kcal) for 3 weeks	*Bacilli* and *Clostridium* clusters XI, XVII, and XVIII (Firmicutes)↑ Bacteroidetes↓.	Adipose tissue↑ weight gain↑ NASH↑
Mice (C57BL/6J) (8 weeks old): healthy male [67]	HFD (60 kcal % fat diet (HFD, D12492) for 8 weeks	Firmicutes↑ *Enterobacteriaceae* (Proteobacteria)↑ Rikenellaceae, *Bacteroidaceae*, and *Provotellaceae* (Bacteriodetes)↓ Ruminococcaceae and *Clostridiales* (Firmicutes)↓ *Proteobacteria* and *Bifidobacterium* (Actinobacteria)↓.	IBD↑ weight gain↑
Mice C57BL/6J (7–10 weeks old): healthy male [68]	Milk fat (rich in palmitic, stearic, myristic, and oleic acids) for 4 weeks	Firmicutes↑ Proteobacteria↑ Actinobacteria↑ Bacteroidetes↓.	Adipose tissue↑ IBD↑ weight gain↑
Mice RELMβ KO (13 weeks old): healthy female [68]	Safflower oil (rich in palmitic acid) for 4 weeks	Firmicutes↑ Tenericutes↑ Actinobacteria↑ Bacteroidetes↓	Adipose tissue↑ IBD↑ weight gain↑
Mice C57BL/6J (3 weeks old): healthy male [69]	HFD palm oil (rich in palmitic acid) with 45% energy for 16 weeks	*Coprococcus*, Erysipelotrichaceae, and Lachnospiraceae (Firmicutes)↑ *Bacteroides*, Bacteroidaceae (Bacteroidetes)↓ Deferribacteres↓ Actinobacteria↓ Proteobacteria↓.	Adipose tissue↑ weight gain↑ insulin resistance↑
Mice (C57BL/6J) (3 weeks old): healthy male [70]	HFD (60% of energy from fat; 95% from lard; and 5% from soybean oil) for 6 weeks	*Desulfovibrio* and *Bilophila wadsworthia* (Proteobacteria)↑ *Bifidobacterium* spp. (Actinobacteria)↓.	Adipose tissue↑ IBD↑
Mice C57BL/6J (6 weeks old): healthy female [71]	HFD saturated fatty acid with 34% energy for 8 weeks	*Lactobacillus*, erysipelotrichaceae, Lachnospiraceae, and *Pseudoflavonifractor* (Firmicutes)↑ *Bilophila* (Proteobacteria)↑ *Allobaculum* (Firmicutes)↓ *Bamesiella* (Bacteroidetes)↓ *Mucispirillum* (Deferribacteres)↓ *Bacteroides* (Bacteroidetes)↓ *Bifidobacterium* (Actinobacteria)↓.	Weight gain↑ adipose tissue↑ insulin resistance↑ IBD↑ gut permeability↑
Mice SPF C57BL/6J (8 weeks old): healthy [72]	HFD with 72% fat/kcal for 9 weeks.	*Clostridium* (Firmicutes)↑ *Bifidobacterium* (Actinobacteria)↓ *Enterococcus* (Firmicutes)↓ *Bacteroides* (Bacteroidetes)↓.	NASH↑
ICR Swiss mice (6 weeks old): healthy male [73]	Butter diet with 38% energy for 12 weeks	*Alistipes indistinctus* (Bacteroidetes)↑ *Marvinbryantia, Lactobacillus* spp. and *Lactococcus* (Firmicutes)↑ *Anaerostipes butyaticus, Desulfovibrio desulfuricans* and *Escherichia fergunsoni* (Proteobacteria)↑ Bacteroidetes↓	Weight gain↑ hypertension↑ insulin resistance↑ total cholesterol↑
Mice C57BL/6N (3 weeks old): healthy female [49]	HFD containing coconut oil 25% and soy oil 0.25% for 2–8 weeks.	*Anaerotruncus, Syntrophomonas, Lutispora* and *Lactobacillus* (Firmicutes)↑ *Parabacteroidetes* (Bacteroidestes)↑ *Akkermansia* (Verruncomicrobia)↑ Proteobacteria↑ *Anaerostipes* and Peptostreptococcaceae (Firmicutes)↓ *Agrobacterium* (Proteobacteria)↓	Obesity↑ adipose tissue↑ plasma cholesterol↑

Abbreviation: ↑ = significant increase; ↓ = significant decrease; IBD = inflammatory bowel diseases; HFD = high-fat diet; LFD = low-fat diet; NASH = nonalcoholic steatohepatitis and ICR = Institute of Cancer Research.

**Table 3:** Effects of monounsaturated fatty acids intake on gut microbiota composition and metabolic outcomes in animal models.

Host	Diets	Main Outcomes	
		Gut Microbiota	Metabolic
Mice C57BL/6J (germ free wild-type): healthy male [98]	Western diet with 41% energy from fat for 8 weeks	Bacteroidetes↑ Firmicutes↓	Adipose tissue↓ obesity↓
Rats: Sprague–Dawley healthy male [99]	LFD 10% (SFA 25.1%, MUFA 34.7%, and PUFA 40.2%) for 8 eight weeks	Bacteroidales (Bacteroidetes)↑ Clostridiales (Firmicutes)↓ Enterobacteriales (Proteobacteria)↓	IBD↓ obesity↓
Mice C57BL/6J (3 weeks old): healthy male [66]	HFD olive oil rich in oleic acid (45% kcal) for three weeks	Bacteroidetes↑ *Bacilli* and *Clostridium* cluster XI, XVII, and XVIII (Firmicutes)↓	Adipose tissue↓ weight gain↓ NASH↓ insulin resistance↓
ICR Swiss mice: 8-week-old healthy female [100]	HFD supplementation with an oleic acid (16% per day) for 19 weeks	Bacteroidetes↑ *Bifidobacterium* spp. (Actinobacteria)↑ *Lactobacillus* spp. (Firmicutes)↓ *Clostridial* cluster XIVa (Firmicutes)↓ Enterobacteriales (Proteobacteria)↓	Obesity↓ IBD↓
Mice C57BL/6J (3 weeks old): healthy male [69]	HFD olive oil (oleic acid) with 45% energy for 16 weeks	*Allobculum*, Erysipelotrichaceae (Firmicutes)↑ *Bacteroides*, Bacteroidaceae (Bacteroidetes)↓ Deferribacteres↓ Proteobacteria↓ Actinobacteria↓	Weight gain↓ NASH↓
Rats (4–5 weeks old): spontaneously hypertensive male [97]	EVOO diet: 20% of EVOO (oleic acid) with 75.5% energy for 12 weeks	Lachnospiraceae, Ruminococcaceae (Clostridia XIVa) and *Lactobacillus* (Firmicutes)↑ Bacteroidetes↓ Actinonobacteria↓	Hypertension↓
ICR Swiss mice (6 weeks old): healthy male [73]	EVOO with 38% energy for 12 weeks	Prevotellaceae, Marinillabiliaceae, *Mucilaginibacter dageonensis, Bacteroides fragilis* and *Alistipes indictintus* (Bacteroidetes)↑ Sutterellaceae and *Marispirillum* (Proteobacteria)↑ Christenellaceae, Erysipelotrichaceae and *Clostridim cocleatum* (Firmicutes)↑ *Desulfovibrio* (Firmicutes)↓	Hypertension↓ weight gain↓

Abbreviation: ↑ = significant increase; ↓ = significant decrease; EVOO = extra virgin olive oil; IBD = inflammatory bowel diseases; HFD = high-fat diet; LFD = low-fat diet; MUFA = medium unsaturated fatty acid; NASH = nonalcoholic steatohepatitis; PUFA = polyunsaturated fatty acid; SFA = saturated fatty acid and ICR = Institute of Cancer Research.

**Table 4:** Effects of monounsaturated fatty acids intake on gut microbiota composition and metabolic outcomes in humans.

Host	Diets	Main Outcomes	
		Gut Microbiota	Metabolic
Men and women volunteers with risk of metabolic syndrome [96]	MUFA-rich oil (canola, 36%; canola/DHA, 39%; and canola oleic, 44% energy) for 4 weeks	*Coprobacillus, Faecalibacterium Lactobacillus, Robinsoniella* and *Tepidimicrobium Fusibacter, Turicibacter* (Firmicutes)↑ *Flexithrix, Parabacteroides*, and *Prevotella* (Bacteroidetes)↑ Enterobacteriaceaes (Proteobacteria)↑ *Isobaculum* (Firmicutes)↓	BMI↓
Men and women obese volunteers with prediabetes risk (≥65 years old) [105]	Lipids 40% (MUFA 19%) for 3 days	*Prevotella* (Bacteroidetes)↓ *Faecalibacterium prausnitzzi*, Lactic acid bacteria (Firmicutes)↑ *Escherichia coli* (Proteobacteria)↑ Firmicutes/Bacteroidetes ratio↑	T2DM↓
Men and women nonobese volunteers with prediabetes risk (≥65 years old) [105]	Lipids 41% (MUFA 19%) for 3 days	Firmicutes/Bacteroidetes↓ *Prevotella* (Bacteroidetes)↑ *Faecalibacterium prausnitzzi*, Lactic acid bacteria (Firmicutes)↑ *Escherichia coli* (Proteobacteria)↓	T2DM↓

Abbreviation: ↑ = significant increase; ↓ = significant decrease; BMI = Body mass index; DHA = docosahexaenoic acids; MUFA = medium unsaturated fatty acid and T2DM = type 2 diabetes mellitus.

**Table 5:** Effects of PUFA intake on gut microbiota composition and metabolic outcomes in animal models.

Host	Diets	Main outcomes	
		Gut microbiota	Metabolic
Mice wild-type (13 weeks old): healthy female [119]	Safflower oil (rich in linoleic acid) for 21 weeks	Clostridiaceae (Firmicutes)↑ Desulfovibrionaceae (Proteobacteria)↑ Bacteriodaceae, Prevotellaceae and Rickenellaceae (Bacteroidetes)↓	Obesity↑
Mice RELMβ KO (13 weeks old): healthy female [119]	Safflower oil (rich in linoleic acid) for 21 weeks	Clostridiaceae (Firmicutes)↑ Desulfovibrionaceae (Proteobacteria)↑ Bacteriodaceae, Prevotellaceae and Rickenellaceae (Bacteroidetes)↓	Obesity↓
Rats: Sprague–Dawley male [99]	LFD 10% (SFA 25%, MUFA 35%, and PUFA 40%) at eight weeks	Bacteroidales (Bacteroidetes)↑ Clostridiales (Firmicutes)↓ Enterobacteriales (Proteobacteria)↓	IBD↓ obesity↓
Mice C57BL/6J (3 weeks old): healthy male [66]	Safflower oil rich in linoleic acid (45% energy) for 8 weeks	Bacteroidetes↑ *Clostridium cluster* XI, XVII, and XVIII (Firmicutes)↑ *Bacilli* (Firmicutes)↓	Adipose tissue↓ obesity↓ NASH↓ insulin resistance↓
Mice C57Bl/6 (7–10 weeks old): healthy male [68]	HFD safflower oil (rich in linoleic acid) for 4 weeks	Firmicutes↑ Tenericutes↑ Actinobacteria↑ Deferibacteria↑ Proteobacteria↑ Bacteroidetes↓	IBD↑ weight gain↑
ICR Swiss mice: 8-week-old healthy female [100]	HFD supplementation with n–3 PUFAs (EPA + DHA) for 19 weeks	*Bifidobacterium* spp. (Actinobacteria)↑ Bacteroidetes↑. *Lactobacillus* spp. (Firmicutes)↑ Enterobacteriales (Proteobacteria)↑ Clostridial cluster XIVa (Firmicutes)↓	IBD↓ Obesity↓
Mice C57BL/6J (24 months old): healthy female [115]	1. HFD of maize oil + rapeseed oil (rich in n–6 PUFAs) with 40% energy for 7 weeks	Firmicutes↑ Bacteroidetes↓	Weight gain↑ IBD↑
	2. LFD of maize oil plus fish oil supplemented (rich in n–3 PUFA (EPA + DHA) with 34% energy for 7 weeks	Bacteroidetes↑ Firmicutes↓ Proteobacteria↓	Weight gain↓ IBD↓
Mice C57BL/6J (3 weeks old): healthy male [69]	1. HFD safflower oil (linoleic acid n–6 PUFA) with 45% energy for 16 weeks	*Allobaculum, Oscillibacter* and Ruminococcaceae (Firmicutes)↑ *Bacteroides* and *Parabacteroides* (Bacteroidetes)↑ *Bifidobacterium* (Actinobacteria)↑	Weight gain↑ Insulin resistance↑
	2. HFD flaxseed/fish oil (α-linolenic acid n–3 PUFA) with 45% energy for 16 weeks	*Allobaculum*, Erysipalotrichaceae and Lachnospiraceae (Firmicutes)↑ Deferribacteres↑ Bifidobacteriaceae (Actinobacteria)↑ *Bacteroides*, Bacteroidaceae (Bacteroidetes)↓ Proteobacteria↓	Weight gain↓ Insulin resistance↓ NASH↓
ICR mice (4 weeks old): healthy male and female (17–21 g) [120]	HFD fish oil (40% EPA and 27% DHA) n–3 PUFA for 2 weeks	*Helicobacter, Pseudomonas* sp., and Sphingomonadales (Proteobacteria)↓ Clostridiales (Firmicutes)↓.	Weight gain↔
Mice BALB/c (3 weeks old): male and female pups from n–3 breeders [121]	HFD n–6/n–3 PUFAs (1/2) with 40% energy for 2 weeks	*Blautia, Oscillibater, Clostridales, Robinsoniella, Lactococcus*, and *Eubacterium* (Firmicutes)↑ Porphyromonadaceae (Bacteroidetes)↓ Lachnospiraceae and *Rosebeuria*, *Euterococcus* (Firmicutes)↓	IBD↓
Mice C57BL/6J (6 weeks old): healthy female [71]	HFD n–3 PUFA with 37% energy for 8 weeks	*Lactobacillus*, *Allobaculum*, *Clostridium*, and *Turicibacter* (Firmicutes)↑ *Bifidobacterium* (Actinobacteria)↑ *Bamesella* (Bacteroidetes)↓ *Bilophila* (Proteobacteria)↓ *Akkemansia* (Verrucomicrobia)↓	Weight gain↓ adipose tissue↓ insulin resistance↓
	HFD n–6 PUFA with 31% energy for 8 weeks	*Allobaculum,* Erysipelotrichaceae, Lachnospiraceae and *Oscillibacter* (Firmicutes)↑ *Mucispitillum* (Deferribacteres)↑ *Bilophila* (Proteobacteria)↑ *Lactobacillus* and *Acetivibrio* (Firmicutes)↓ *Bamesiella* (Bacteroidetes)↓ *Bifidobacterium* (Actinobacteria)↓	Weight gain↑ adipose tissue↑ insulin resistance↑ IBD↑
Rats (5 weeks old): early life stressed (weaned) female pups (250–300 g) with reduced Bacteroidetes/ Firmicutes ratio and inflamed gut [122]	HFD of n–3 PUFA (1 g EPA 80% + DHA 20%) for 17 weeks	*Butyrivibrio*, *Jeotgalicoccus*, and *Peptococcus* (Firmicutes)↑ *Caldicoprobacter* (Terrabacteria)↑ *Bifidobacteria* and *Aerococcus* (Actinobacteria)↑ *Undibacterium* (Proteobacteria)↓	IBD↓
Mice C57BL/6J (4–5 weeks old) and adulthood (11–13 weeks old): male offspring subsequently weaned onto the same diets as their mothers and stressed. Stressed adulthood [123]	HFD of n–3 PUFA-supplemented diet (1 g EPA + DHA/100 g diet) for 8 weeks	Bacteroidetes↑ *Verrucomicrobia* and *bifidobacterium* (Actionobacteria)↑ Firmicutes↓ *Tenercutes* and *enterobacteria* (Proteobacteria)↓	IBD↓
Mice C57BL/6 WT (4 weeks old): transgenic male and female lactated by mother lactated or foster mother [124]	Maternal n–3 PUFA for 4 weeks plus HFD 60% energy (SFA, 32%; MUFA, 36%; PUFA, 32%; n–6 PUFA, 30%; and n–3 PUFA, 2.1%) for six weeks	*Helicobacter* (Proteobacteria)↑ *Bacteroides* (Bacteroidetes)↑ *Epsilonproteobacteria* (Proteobacteria)↑ Lachnospiraceae and Ruminococcaceae (Firmicutes)↑ *Akkermansia* (Verrucomicrobia)↑	Obesity↓ IBD↓
Rats with diabetes mellitus (7 weeks old): male and female with type 2 diabetes mellitus [80]	1. LFD n–6/n–3 (3/1) for 6 weeks 2. HFD with n–6/n–3 (9/1) for 6 weeks	Proteobacteria↑ *Allobaculum* (Firmicutes)↑ Actinobacteria↓ Firmicutes/Bacteroidetes↓	Weight gain↓ IBD↓ insulin resistance↓ T2DM↓

Abbreviation: ↑ = significant increase; ↓ = significant decrease; ICR = Institute of Cancer Research; IBD = inflammatory bowel diseases; HFD = high-fat diet; DHA = docosahexaenoic acid; EPA = eicosapentaenoic acid; LFD = low-fat diet; MUFA = medium unsaturated fatty acid; NASH = nonalcoholic steatohepatitis; PUFA = polyunsaturated fatty acid; SFA = saturated fatty acid and T2DM = type 2 diabetes mellitus.

**Table 6:** Effects of PUFA intake on gut microbiota composition and metabolic outcomes in humans.

Host	Diets	Main Outcomes	
		Gut Microbiota	Metabolic
Men and women (young): 98 healthy volunteers [135]	HFD n–3 PUFA	Bacteroidetes↑ Actinobacteria↑ Firmicutes↓ Proteobacteria↓	Weight gain↓
Men (45 years old): healthy and physically active [118]	Fish protein diet with vegetables that included over 600 mg of HFD n–3 PUFA for 2 weeks	*Blautia, Coprococcus, Ruminococcus, Subdoligranulum, Eubacteria, Anaerosfipes*, and *Pseudobutyrivibrio* (Firmicutes)↑ *Roseburia* and *Faecalibacterium prausnitzii* (Firmicites)↓ *Akkermansia* spp. (Verrucomicrobia)↓ Bacteroidetes↓ Actinobacteria↓	IBD↓ T2DM↓ obesity↓ insulin resistance↓
Men and women: volunteers with risk of metabolic syndrome [96]	HFD n–6 PUFA blended corn/safflower oil (25/75) with 42% energy and blended flax/safflower oil (6/4) with 42% energy for 4 weeks	*Isobaculum* (Firmicutes)↑ *Parabacteroides* and *Prevotella*, Bacteroidetes↓ Enterobacteriaceae↓ Turicibacter (Firmicutes)↓	BMI↓
Women twins (middle and elderly aged): 876 healthy [101]	HFD in n–6/n–3 PUFA (11/1) for 7 days	Lachnospiraceae (Firmicutes)↑	BMI↓ obesity↓
Men and women (≥50 years old): healthy [102]	Capsules and drink of n–3 PUFA (EPA + DHA) for 8 weeks	*Bifidobacterium* (Actinobacteria)↑ *Oscillospira*, *Roseburia* and *Lachnospira* (Firmicutes)↑ *Coprococcus* and *Faecalibacterium* (Firmicutes)↓	BMI↓

Abbreviation: ↑ = significant increase; ↓ = significant decrease; BMI = body mass index; IBD = inflammatory bowel diseases; HFD = high-fat diet; DHA = docosahexaenoic acid; EPA = eicosapentaenoic acid; PUFA = polyunsaturated fatty acid and T2DM = type 2 diabetes mellitus.
